# Molecular Variation at a Candidate Gene Implicated in the Regulation of Fire Ant Social Behavior

**DOI:** 10.1371/journal.pone.0001088

**Published:** 2007-11-07

**Authors:** Dietrich Gotzek, D. DeWayne Shoemaker, Kenneth G. Ross

**Affiliations:** 1 Department of Ecology and Evolution, University of Lausanne, Lausanne, Switzerland; 2 United States Department of Agriculture–Agricultural Research Service (USDA-ARS) Center for Medical, Agricultural, and Veterinary Entomology, Gainesville, Florida, United States of America; 3 Department of Entomology, University of Georgia, Athens, Georgia, United States of America; University of Edinburgh, United Kingdom

## Abstract

The fire ant *Solenopsis invicta* and its close relatives display an important social polymorphism involving differences in colony queen number. Colonies are headed by either a single reproductive queen (monogyne form) or multiple queens (polygyne form). This variation in social organization is associated with variation at the gene *Gp-9*, with monogyne colonies harboring only *B*-like allelic variants and polygyne colonies always containing *b*-like variants as well. We describe naturally occurring variation at *Gp-9* in fire ants based on 185 full-length sequences, 136 of which were obtained from *S. invicta* collected over much of its native range. While there is little overall differentiation between most of the numerous alleles observed, a surprising amount is found in the coding regions of the gene, with such substitutions usually causing amino acid replacements. This elevated coding-region variation may result from a lack of negative selection acting to constrain amino acid replacements over much of the protein, different mutation rates or biases in coding and non-coding sequences, negative selection acting with greater strength on non-coding than coding regions, and/or positive selection acting on the protein. Formal selection analyses provide evidence that the latter force played an important role in the basal *b*-like lineages coincident with the emergence of polygyny. While our data set reveals considerable paraphyly and polyphyly of *S. invicta* sequences with respect to those of other fire ant species, the *b*-like alleles of the socially polymorphic species are monophyletic. An expanded analysis of colonies containing alleles of this clade confirmed the invariant link between their presence and expression of polygyny. Finally, our discovery of several unique alleles bearing various combinations of *b*-like and *B*-like codons allows us to conclude that no single *b*-like residue is completely predictive of polygyne behavior and, thus, potentially causally involved in its expression. Rather, all three typical *b*-like residues appear to be necessary.

## Introduction

A main goal of evolutionary genetics is to document naturally occurring variation and to reconcile observed patterns with population history and demography, fitness consequences, and selection regimes at genes of interest [1], [2]. Study of the adaptive maintenance of molecular variation historically has followed one of two approaches, elucidation of the functional components of molecular adaptations at the biochemical level, or description of the historical footprints of selection acting on sequence variants [3]. An important objective in modern studies of molecular adaptation is to bridge the two approaches by means of comprehensive research integrating functional data with information on patterns of variation that implicate past selection [4] (see also [3], [5]–[7]).

The fire ant *Solenopsis invicta* displays an important colony-level social polymorphism that is associated with variation at a single gene, *general protein-9* (*Gp-9*) [8]. Colonies with a single reproductive queen (monogyne colonies) always feature the exclusive presence of *B*-like alleles of *Gp-9* in all colony members. In contrast, colonies with multiple reproductive queens (polygyne colonies) always have an alternate class of alleles, designated *b*-like alleles, represented along with *B*-like alleles among colony members [8]–[11]. This pattern, coupled with similar genetic compositions of monogyne and polygyne populations at numerous other nuclear genes, has led to the hypothesis that the presence of *b*-like alleles in a colony's workers is both necessary and sufficient to elicit polygyne social behavior [8]–[10], [12], [13]. Because variation in queen number represents a fundamental social polymorphism that is associated with a suit of important reproductive, demographic, and life history differences [14]–[16], variation at *Gp-9* is hypothesized to underlie the expression of major alternative adaptive syndromes in *S. invicta*.

Our understanding of the association of variation at *Gp-9* with colony social organization has been advanced by the production of sequence data for *S. invicta* in its native (South American) and introduced (USA) ranges as well as for numerous other *Solenopsis* species [10], [11]. These studies revealed several important patterns. First, the monophyletic *b*-like alleles are restricted to a clade of six South American fire ant species that display the monogyne-polygyne polymorphism (this group of species, which includes *S. invicta*, is informally termed the socially polymorphic clade). Second, polygyne colonies in all of the socially polymorphic species always contain *b*-like alleles. Third, the *b*-like alleles of the socially polymorphic species bear diagnostic amino acid residues at positions 42, 95, and 139 that distinguish them from all other *Gp-9* alleles (collectively known as *B*-like alleles). This finding prompted speculation that the substitutions at one or more of these positions may alter the function of GP-9 protein with respect to its proposed role in modulating social behavior [10], [11]. Finally, the *b* alleles, which comprise a small clade of *b*-like alleles that apparently arose recently in *S. invicta*, feature a radical, charge-changing substitution (Glu151Lys) that may underlie their observed deleterious effects in homozygous condition [17].

GP-9 protein is a member of the insect odorant-binding protein family [10]. Several well studied proteins in this family have been implicated as important molecular components of chemoreception in insects, presumably effecting the transduction of pheromones or food chemostimulants to neuronal signals by transporting these ligands through the chemosensillar lymph to neuronal receptors [18]. Regulation of colony queen number in fire ants involves reciprocal chemical signalling and perception between workers and queens, with workers ultimately making decisions about which queens, and how many, are tolerated as colony reproductives based on queen pheromonal signatures [9], [19]. The role of some odorant-binding proteins in chemoreception, the invariant association of one class of *Gp-9* alleles with polygyny, and the restriction of this class of alleles to the socially polymorphic clade of South American fire ants have been viewed as evidence that *Gp-9* may directly influence social organization rather than merely being a marker for other genes of major effect on this trait (reviewed in [20]).

Further complexities in our understanding of *Gp-9* and fire ant social evolution have arisen as additional sequence data have been generated. For instance, it is now apparent that variation at this gene is not invariably associated with colony social organization in the genus *Solenopsis*, given that the fire ant *S. geminata*, a distant North American relative of *S. invicta*, does not exhibit allelic variation associated with colony social form [21]. Also, discovery of a *Gp-9* allele with a *b*-like amino acid residue at position 95 but *B*-like residues at positions 42 and 139 in the undescribed *S.* species “X” indicates that the two classes of alleles in the socially polymorphic species are not as internally homogeneous as previously believed [11]. Unfortunately, the social organization of the source colony for this sequence could not be determined, precluding a test of the importance of residue 95 in regulating queen number. These recent results suggest that progress in our understanding of *Gp-9* in fire ants has been hampered by limited knowledge of naturally occurring sequence variation; in even the best studied species, *S. invicta*, only a handful of individuals from a single site in the native range have been sequenced to date.

To remedy this shortcoming, the present study documents sequence variation at *Gp-9* using extensive samples of *S. invicta* collected over a large portion of its vast native range as well as samples of many of its fire ant relatives. Few previous studies have employed such large collections of intraspecific sequence variants in order to analyze patterns of adaptive molecular variation at single genes [22]. Our specific objectives were to characterize the molecular evolution of *Gp-9* in fire ants, to test for effects of selection on the gene, to confirm the association between polygyny and the presence of variants encoding *b*-like amino acid residues, and to test for phylogeographic patterns in the distribution of the observed variation in *S. invicta*. We were particularly interested in finding new variants encoding unique combinations of *B*-like and *b*-like residues at codons 42, 95, and 139, with the hope that their discovery might shed light on the role of each substitution in mediating the expression of social organization. In combination with the other analyses, information from such variant colonies is expected to aid progress in connecting the genetic and phenotypic variation underlying regulation of fire ant social behavior.

## Results

### General results

**Figure 1:**
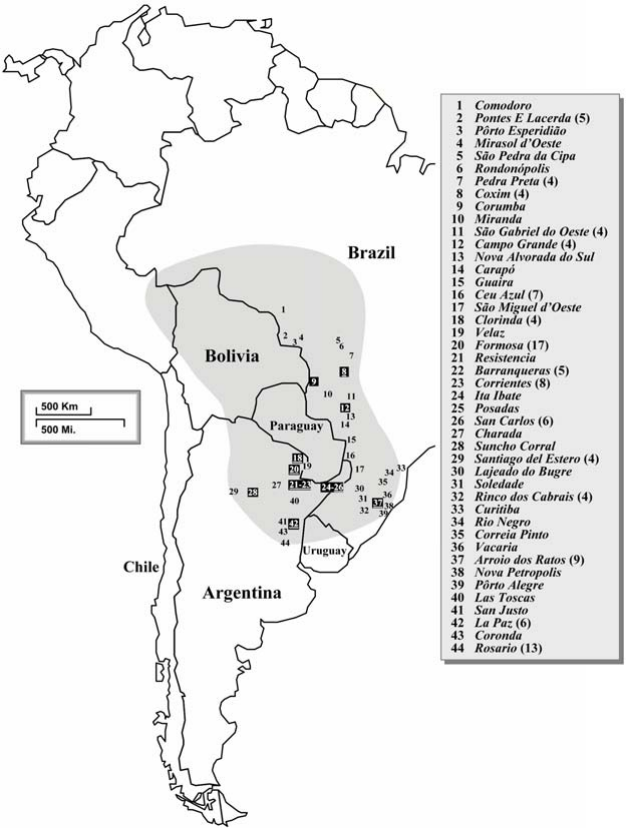
Locations of sampling sites for *S. invicta* in South America. The native range of the species is indicated by gray shading on the map. The number of nests sampled at a site ( = number of sequences obtained) is indicated in parentheses in the key to the sites if greater than one. Sites at which *b*-like alleles were found are highlighted with black rectangles; those at which the *b* alleles of the *b*-like class were found are underlined.

**Figure 2:**
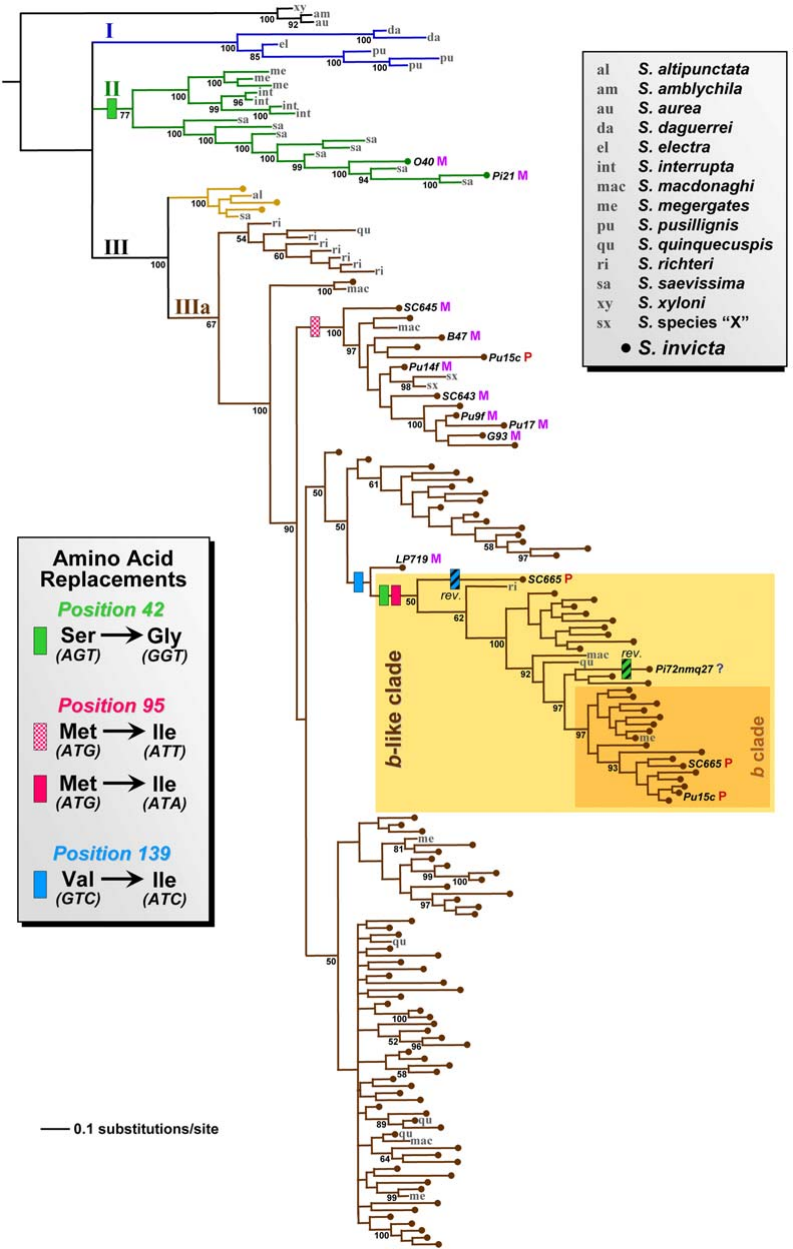
Summary hypothesis of phylogenetic relationships of fire ant *Gp-9* sequences based on Bayesian inference. Sequences from species other than *S. invicta* are indicated by specific abbreviations in gray. Sequences from *S. invicta* are indicated by dots; the identity and social form (M, monogyne; P, polygyne; ?, unknown) of the colony of origin are also indicated for critical sequences. All colonies from which alleles of the *b*-like clade were recovered were confirmed to be polygyne. Inferred amino acid replacements at three codons considered to be diagnostic for *b*-like alleles are mapped onto the phylogeny (reversals are indicated by bars with cross-hatching). Posterior clade probabilities >50% are indicated as percentages below branches.

The complete data set consisted of 185 full-length *Gp-9* sequences (149 newly generated), of which 136 were from the focal species, *S. invicta* (sampling sites for this species are shown in Figure 1; GenBank accession numbers for all sequences are listed in Table S1). A total of 164 unique sequences were identified, of which 121 were recovered from *S. invicta*. This is a large increase over the six alleles previously described from this species based on sampling at a single locality in Argentina and throughout the introduced USA range [10], [11]. The great majority of *Gp-9* alleles in the complete fire ant data set (91%) were represented as singletons. Among the 13 alleles recovered from more than one specimen, three were shared between *S. invicta* and ants identified as belonging to the closely related species *S. quinquecuspis* or *S. megergates* (see Figure 2).

Plots of transition and transversion rates against pairwise sequence distances for various sets of alleles revealed little evidence of mutational saturation in the non-coding regions or at any of the three codon positions in exons (data not shown). For the entire data set, a consistent A+T bias in base composition was found, which is more substantial in the non-coding regions (0.816) than coding regions (0.574). No evidence of non-random codon usage was found over the entire set of sequences (scaled χ^2^ = 0.104 using Yates correction; effective number of codons = 57.07 out of a maximum of 61; codon bias index = 0.348). Also, there is no evidence for intragenic recombination having occurred (all potential recombination events fall below 95^th^ percentile significance level [DSS: 84%; LRT: 46%]). This latter result parallels the lack of evidence for recombination at *Gp-9* reported by Krieger and Ross [11].

### Phylogenetic relationships of *Gp-9* alleles

All phylogenetic hypotheses recovered by the differing methods are compatible with one another. However, the Shimodaira and Hasegawa (SH) test identified the relatively poorly resolved maximum parsimony (MP) tree as significantly worse than the Bayesian inference (BI) tree (Δ-lnL = 149.233, *P* = 0.015), whereas the minimum evolution (ME) and BI trees did not differ significantly (Δ-lnL = 36.944, *P* = 0.376). The poor overall node resolution obtained with the MP analysis is consistent with the relatively few parsimony-informative sites (134) among the 164 unique sequences. The relatively high standard deviations of split frequencies in the BI analyses (∼0.085) also suggest limited information content of our data set, which results in an inability to resolve some parts of the phylogeny with confidence (e.g., [23]).

The phylogenetic hypothesis produced by BI is shown in Figure 2. Three major *Gp-9* allele clades of unresolved relationship to one another are apparent among the ingroup sequences (those recovered from the South American fire ants). Clade I contains sequences from three *Solenopsis* species considered to be rather distant relatives of *S. invicta* [24]. Clade II contains sequences from an assortment of species more or less closely related to *S. invicta*, as well as two *S. invicta* sequences. Clade III contains two relatively well supported lineages. One includes three *S. invicta* sequences along with sequences from two quite distant relatives (*S. altipunctata* and *S. saevissima*). The second, large lineage (Clade IIIa) contains only *Gp-9* alleles recovered from the socially polymorphic South American fire ants (*S. invicta* and its close relatives *S. richteri*, *S. megergates*, *S. quinquecuspis*, *S. macdonaghi*, and *S.* species “X”). The relationships of the major *Gp-9* lineages depicted in Figure 2 mirror the relationships inferred by Krieger and Ross [11] from a much smaller data set. Importantly, the previously detected paraphyly and polyphyly of *S. invicta Gp-9* alleles with respect to those of the other socially polymorphic species [10], [11] are extended here to even more distantly related fire ant species.

Within the lineage comprising exclusively alleles from the socially polymorphic species (Clade IIIa), a clade composed almost entirely of sequences from *S. richteri* forms a sister lineage to the remaining sequences. This evidence for a basal position of *S. richteri* alleles within the group of alleles of all socially polymorphic species again is consistent with earlier conclusions based on smaller data sets [10], [11].

Significantly, the *b*-like allele clade is recovered once again in our study, confirming a monophyletic origin of the variants associated with polygyny in the South American fire ants. Support for such a clade varies depending on how it is defined. If defined only by possession of the Ile^139^ residue (the first of the three substitutions typical of *b*-like alleles to appear in the lineage), the clade is supported by a posterior probability value <0.5. However, if defined by possession of all three residues considered diagnostic for the *b*-like allele class, the posterior probability value increases to 0.5. (Note that the Val^139^ encoded by a colony SC665 sequence apparently represents a reversal; Figure 2). Given the evidence that sequences encoding all three residues are required for the expression of polygyny (below), we choose to define the *b*-like clade by the synapomorphy of joint possession of residues Gly^42^ and Ile^95^, thus excluding from the group the sequence from colony LP719 encoding Ile^139^ but neither of these other residues (see Figure 2).

Finally, our data verify that the *b* alleles of *S. invicta* form a relatively recently derived monophyletic group within the *b*-like clade. This confirms earlier evidence that the radical, charge-changing Glu151Lys replacement characterizing these alleles occurred only once, presumably in an ancestral *S. invicta* population from northeastern Argentina or southeastern Brazil, where the alleles currently predominate (see Figure 1).

### Nucleotide variation at *Gp-9*

Most nucleotide sites (85%) are invariant across all *Gp-9* sequences from the different *Solenopsis* species, and the two most divergent sequences (from *S. aurea* and *S. invicta*) differ at just 47 (2.1%) of their nucleotides. Most of the variable sites in the total collection of sequences (66%) occur in the non-coding regions. However, because these regions encompass 80% of the total sequence length, proportionately more variable sites occur in the coding regions (22%; 21% for third codon positions) than in the non-coding regions (14%).

**Figure 3:**
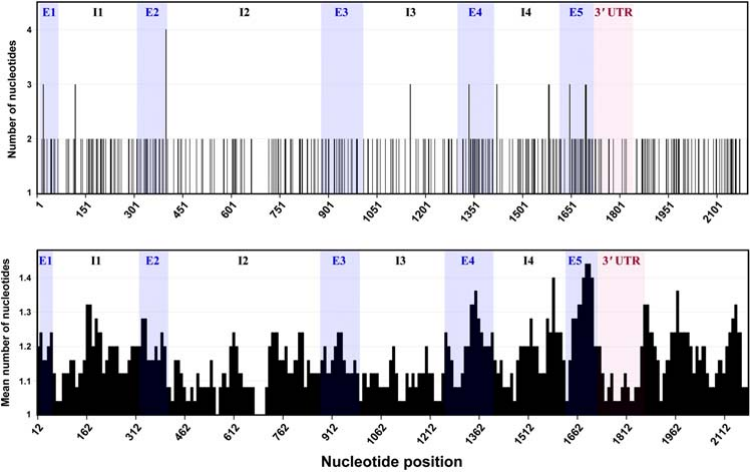
Nucleotide variation along *Gp-9* and its 3′ flanking region in all *Solenopsis* study species. The top graph shows the number of different nucleotides at each position; the bottom graph shows the mean number in a 25-bp sliding window moved by 10-bp increments. Blue shading indicates approximate positions of exons (E1-E5) and pink shading indicates the 3′-UTR. Introns are designated by I1-I4.

The magnitude of variation at various positions along *Gp-9* and its 3′ flanking region in all the study species is shown in Figure 3. This depiction of per-site nucleotide counts confirms that overall variation is not conspicuously lower in the exons than in the non-coding regions; indeed, the average number of different nucleotides per site is 1.23 in the exons (all sites as well as third codon positions) but only 1.14 in the non-coding regions. However, considerable heterogeneity exists within both types of regions. For instance, a notable spike in variation is evident in the 3′ portion of exon 5, with 34% of nucleotide positions 1646-1708 (33% of the third codon positions) comprising variable sites (these sites correspond to amino acid residues 134-153). On the other hand, conspicuous dips in variation are apparent at nucleotide positions 620-715 in intron 2 (5% variable sites) and in the 3′-UTR (3′-untranslated region; 6% variable sites). Very similar patterns in the distribution of variation along *Gp-9* were observed when just the sequences recovered from the socially polymorphic species or from *S. invicta* were considered (data not shown).

**Figure 4:**
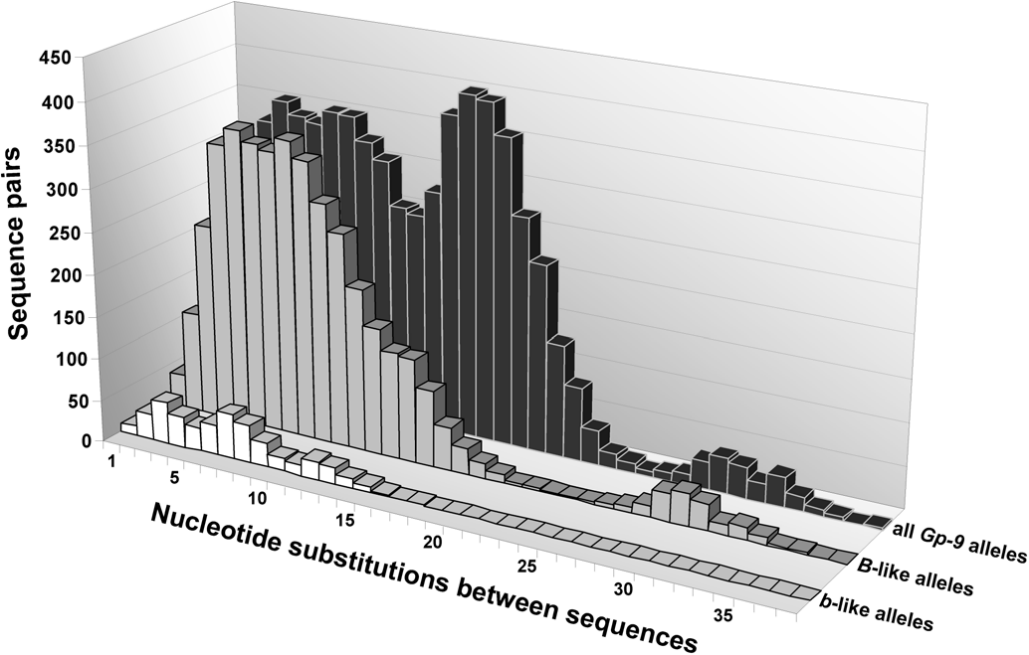
Distributions of numbers of nucleotide substitutions between pairs of unique *Gp-9* sequences from *S. invicta.*

The great majority of unique alleles from *S. invicta* are highly similar to one another at the nucleotide sequence level; indeed, most alleles within the *B*-like and *b*-like classes differ by fewer than a dozen point substitutions (Figure 4). The two most divergent *B*-like alleles differ at only 36 of their nucleotides (1.5%), while the two most divergent *b*-like alleles differ at half that number. Considering both classes combined, an additional peak at 15–20 substitutions attributable to differences between *B*-like and *b*-like alleles becomes apparent (Figure 4).

Several diversity statistics for *Gp-9* from *S. invicta* are presented in Table 1. The mean number of substitutions between pairs of alleles (*d*) varies from less than one for third codon positions in *b*-like alleles to almost seven in non-coding regions when all alleles are combined. In parallel with the observed patterns of site variation across sequences from all the study species, the mean proportions of substitutions (*p*) between *S. invicta* coding-regions consistently exceed those between non-coding regions, regardless of whether all codon positions or just third positions are considered. While this difference between the coding and non-coding regions is marginally non-significant based on a Fisher's exact test (*P* = 0.071), a phylogeny-based resampling test showed that coding-region tree lengths always were considerably greater than any of the tree lengths derived from non-coding regions, under both MP and maximum likelihood (ML) criteria (thus, substitution rates in coding regions exceed those in non-coding regions at *P*<0.001). Considering only the *b*-like alleles of *S. invicta*, a greater than 3-fold excess of coding-region substitutions exists, attributable mostly to augmented variation in the first and second codon positions. Remarkably, given the phylogenetic restriction of the *b*-like clade and the fact that only 1/3 as many *b*-like as *B*-like *S. invicta* sequences were studied, values of both *d* and *p* over all codon positions are greater for the *b*-like group. These patterns hint at the possibility that positive selection has driven the molecular evolution of *Gp-9* in the *b*-like clade.

**Table 1:** Diversity statistics for *Gp-9* from *S. invicta*.

	*d* a			*p* b						
		Coding regions			Coding regions					
	Non-coding regions	All codon positions	Third codon positions	Non-coding regions	All codon positions	Third codon positions	Prop. variable codons	Prop. variable amino acids	Codon diversity	Amino acid diversity
*b*-like alleles	3.62	3.32	0.41	0.0021	0.0072	0.0027	0.1242	0.0980	0.0253	0.0221
*B*-like alleles	6.20	3.05	1.26	0.0036	0.0067	0.0082	0.3766	0.3052	0.0271	0.0211
all *Gp-9* alleles	6.66	5.41	1.76	0.0039	0.0118	0.0109	0.4351	0.3571	0.0362	0.0308

a Uncorrected mean number of nucleotide substitutions between all pairs of unique alleles [56]

b Uncorrected mean proportion of nucleotide substitutions between all pairs of unique alleles [56]

Apart from point substitutions, a single previously unknown structural change in *Gp-9* also was detected. A sequence from an *S. invicta* colony in Santiago del Estero, Argentina, carries a unique point mutation in exon 5 that transforms the stop codon (TAA) at position 154 into a glutamine-encoding codon (CAA), thereby extending the C-terminal tail of the resulting protein by 22 amino acids.

### Transition bias at *Gp-9*

Ratios of the rates of transitions to those of transversions in the coding regions are shown for several data subsets in Table 2. Transition bias is negligible and similar between the third codon position and positions 1+2 for the *B*-like alleles. In contrast, a huge disparity in transition bias exists between these codon positions in the *b*-like alleles, due to the combined effects of a modest bias towards transitions at positions 1+2 and a sharp bias towards transversions at the third position. Over all studied sequences, there is a slight transition bias at the first two codon positions and a negligible bias at the third. These results suggest different patterns of selection acting on *B*-like and *b*-like alleles; because the great majority of third position transversions are nonsynonymous, their elevated rates only in the *b*-like clade are consistent with positive selection having acted specifically on this lineage (see [25]).

**Table 2:** Transition/transversion rate ratios (transition bias) for *Gp-9* coding regions in *Solenopsis*.

		Codon position	
Class of *Gp-9* sequences		1+2	3
all *Solenopsis* sequences		3.01	1.30
Clade IIIa sequences	*b*-like	3.14	0.09
	*B*-like	1.23	0.81
*S. invicta* sequences	*b*-like	3.30	0.11
	*B*-like	1.07	1.22

### Amino acid replacements at *Gp-9*

**Figure 5:**
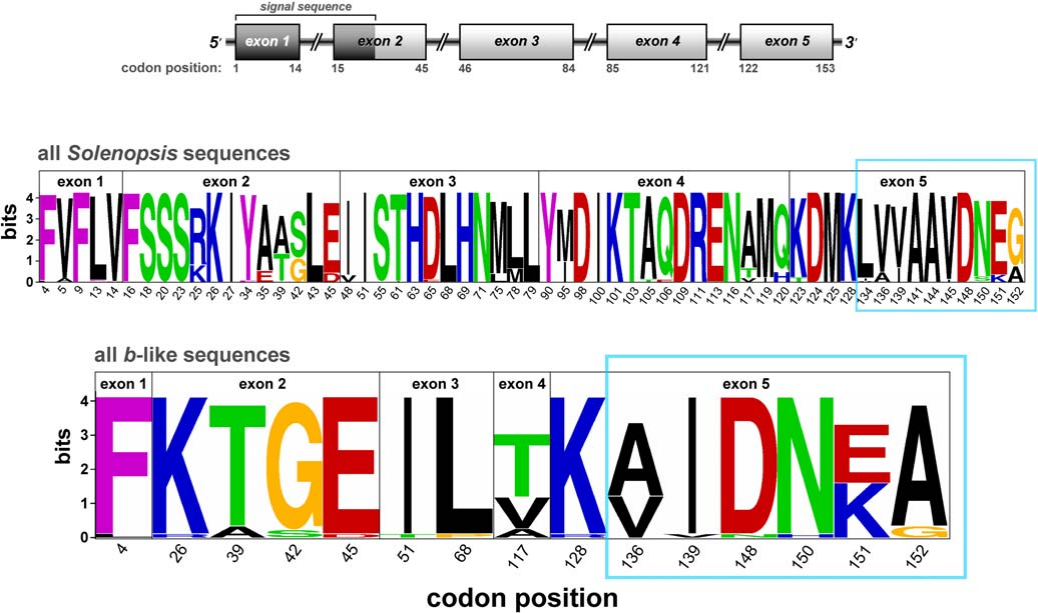
Sequence logos for variable amino acids encoded by unique *Gp-9* sequences in all *Solenopsis* study species and in the *b*-like clade. Logos represent each position by a stack of letters, with the height of each letter proportional to the frequency of the amino acid in the recovered sequences. Overall stack height is proportional to the sequence conservation at that position (measured in bits, maximum sequence conservation is 4.3 bits). A schematic of the exon/intron structure of fire ant *Gp-9* is shown above the logos. Codons in the highly variable 3′ portion of exon 5 are demarcated with light blue boxes.

The sequence logo depicting the variable amino acids encoded by *Gp-9* from all the study species (Figure 5) reveals that the great majority of polymorphic positions feature a single dominant residue along with a second minor residue represented in just one or two sequences. A very similar pattern is seen for sequences from just Clade IIIa or from just *S. invicta*, although several additional positions are monomorphic in each of these smaller data sets (data not shown). Major polymorphisms found in all the data sets at positions 39, 42, 117, 120, 151, and 152 feature substitutions between amino acids of different property groups. All of these positions except 151 also are implicated to be under positive selection by comparison of nonsynonymous and synonymous substitution rates (below). Position 42 is of special note because it features one of the three replacements defining the *b*-like allele clade.

A surprisingly large number of amino acid replacements appear to have occurred during the evolution of the *b*-like clade, based on parsimony reconstruction. The three jointly diagnostic positions, 42, 95, and 139, feature replacements at or near the base of the clade (see Figure 2). Also, position 39 underwent one replacement at the base of and one within the clade, while position 117 underwent five replacements within the clade. Finally, positions 136, 151, and 152 underwent single replacements within the clade. Several of these replacements involve codons in the 3′ portion of exon 5 (Figure 5), thus paralleling the spike in nucleotide variation observed in this region (Figure 3).

Within *S. invicta*, over 43% of codons and 35% of amino acid residues are variable across all recovered alleles (Table 1), values that drop minimally for the *B*-like alleles but substantially for the *b*-like alleles. The similar values for the two metrics within each allele class suggest that the great majority of coding-region nucleotide substitutions have yielded amino acid replacements, a conclusion reinforced by the similarities between the codon diversity and amino acid diversity estimates. The prevalence of nonsynonymous substitutions is reflected also in the similar or higher estimates of *p* from all codon positions relative to third codon positions (Table 1). A similar prevalence of nonsynonymous coding-region substitutions exists across all the species; about 90% of codons exhibit identical codon and amino acid diversities, indicating that every observed nucleotide substitution at these locations caused an amino acid replacement.

An important finding of our survey is the existence of considerable amino acid variation across the three *Gp-9* codons regarded as jointly diagnostic for the *b*-like and *B*-like allele classes in the socially polymorphic species. Alleles of the *b*-like class were reported earlier to always encode Gly^42^, Ile^95^, and Ile^139^ residues, whereas alleles of the *B*-like class generally were found to encode Ser^42^, Met^95^, and Val^139^ residues [10], [11]. We recovered 17 sequences from native *S. invicta* that feature some combination of *b*-like residues at one or two of these crucial positions and *B*-like residues at the remaining position(s). Two *S. invicta* sequences in Clade II (from colonies O40 and Pi21) bear the *b*-like Gly^42^ residue together with the *B*-like Met^95^ and Val^139^ residues (Figure 2); remarkably, the Ser42Gly replacement characterizing this clade apparently occurred independently of the analogous replacement at the base of the *b*-like clade. A well supported group within Clade IIIa includes 12 *S. invicta* sequences encoding the *b*-like Ile^95^ residue together with the *B*-like Ser^42^ and Val^139^ residues; again, the Met95Ile replacement at the base of this group occurred independently of the analogous replacement in the stem lineage of the *b*-like clade (Figure 2). A sequence from colony LP719 encodes the *b*-like Ile^139^ residue together with the *B*-like Ser^42^ and Met^95^ residues. Finally, two *S. invicta* sequences within the *b*-like clade experienced apparent reversals from a *b*-like to *B*-like residue, at position 42 (colony Pi72nmq27) or position 139 (colony SC665) (Figure 2). The significance of these novel *Gp-9* sequences with respect to the form of social organization expressed by the source colonies is explained next.

### Association of polygyny with b-like residues in S. *invicta*

All 28 *S. invicta* colonies from which alleles encoding all three characteristic *b*-like residues were recovered were shown by microsatellite analysis to be polygyne (i.e., they contained multiple offspring matrilines). On the other hand, ten exemplar colonies yielding sequences representing all the major *B*-like clades were shown to be monogyne (i.e., they contained only a single offspring matriline). Thus, we further confirm an important conclusion from previous limited surveys in the native range [10], [26]; possession of typical *b*-like alleles by a colony's workers invariably is linked to the expression of polygyne social organization.

We also were able to determine the social form of 12 of the 17 *S. invicta* colonies that yielded novel *Gp-9* variants encoding some combination of *b*-like and *B*-like residues at positions 42, 95, and 139. Data from the first ten such colonies listed in Table 3, all of which were monogyne, reveal that no single *b*-like residue is associated with polygyny. Significantly, the two polygyne colonies found to contain such novel variants (Pu15c, SC665) were found upon further sequencing of additional colony members to also contain workers with typical *b*-like alleles. Based on these data, we conclude that all three characteristic *b*-like residues may be jointly required for the expression of polygyny in the socially polymorphic South American fire ants.

**Table 3:** Association of colony social organization in *S. invicta* with amino acid residues at three *Gp-9* codons jointly diagnostic for *B*-like and *b*-like alleles.

Colony	Colony social organization^a^	Codon^b^		
		42	95	139
O40	monogyne	***b*** **-like**	*B*-like	*B*-like
Pi21	monogyne	***b*** **-like**	*B*-like	*B*-like
SC645	monogyne	*B*-like	***b*** **-like**	*B*-like
B47	monogyne	*B*-like	***b*** **-like**	*B*-like
Pu14f	monogyne	*B*-like	***b*** **-like**	*B*-like
SC643	monogyne	*B*-like	***b*** **-like**	*B*-like
Pu9f	monogyne	*B*-like	***b*** **-like**	*B*-like
Pu17	monogyne	*B*-like	***b*** **-like**	*B*-like
G93	monogyne	*B*-like	***b*** **-like**	*B*-like
LP719	monogyne	*B*-like	*B*-like	***b*** **-like**
Pu15c^c^	**polygyne**	*B*-like	***b*** **-like**	*B*-like
		***b*** **-like**	***b*** **-like**	***b*** **-like**
SC665^c^	**polygyne**	***b-like***	***b-like***	*B-like*
		***b*** **-like**	***b*** **-like**	***b*** **-like**

*b*-like residues and polygyne social organization are shown in bold for emphasis.

a Determined by microsatellite analysis

b *B*-like residues are Ser^42^, Met^95^, and Val^139^, whereas *b*-like residues are Gly^42^, Ile^95^, and Ile^139^

c Two different sequences were recovered from workers in these colonies; in each case, one represents a typical *b*-like allele (see Figure 2)

### Selection on *Gp-9*

All of the selection analyses we undertook yielded evidence of selection of some form at various codons in *Gp-9* and on various branches of the allele phylogeny. Among the site-specific methods, the single likelihood ancestor counting (SLAC) method identified two positively selected codons (Table 4), but the Suzuki-Gojobori counting method identified none. The former method also detected negative selection on codon 32, while both methods detected such selection on codon 99 (the two positions are invariant in the complete data set). The Bayesian random-effects method detected ten positively selected positions with very high confidence (posterior probability >95%) and another four with less confidence (posterior probability 90–95%) (Table 4). (A tendency of the counting methods to produce more conservative results than the random-effects method has been reported previously [27].) Three of the 14 positions implicated by the latter method as being positively selected (134, 145, and 152) are in the highly variable 3′ portion of exon 5 (Figures 3 and 5).

**Table 4:** Site-specific positive selection on *Gp-9* in *Solenopsis* identified by different methods.

Codon	Method		
	Site-specific		Branch-site-specific
	SLAC counting^a^ *(dN*-*dS*, *P)*	Bayesian random-effects *(dN*/*dS*, post. prob.^b^ *)*	Bayes empirical Bayes random-effects^c^ *(dN*/*dS*, post. prob.*)*
39	2.49, 0.13	79, **1.00**	17.5, 0.957
42	―	79, **0.986**	―
45	―	79, **0.953**	―
48	―	79, **0.973**	―
61	―	79, 0.935	―
75	―	79, **0.995**	―
78	―	79, **0.998**	―
95	―	79, **0.993**	―
117	2.50, 0.13	79, **1.00**	17.5, 0.995
119	―	79, 0.900	―
120	―	79, **0.973**	―
134	―	79, **0.998**	―
145	―	79, 0.907	―
152	―	79, 0.908	―

Dashes indicate the absence of evidence for positive selection using a particular method.

a A nominal α-level of 0.25 is used to indicate statistical significance of selection for this method [27]

b Posterior probabilities >95% obtained using this method are shown in bold

c Only branches of the *b*-like clade were examined for positive selection using this method

Considering branch-specific selection, the counting method of Zhang et al. [28] revealed evidence of positive selection along three branches of the *Gp-9* phylogeny. Two of these are the stem lineage and its succeeding descendant branch at the base of the *b*-like radiation (*d*_N_/*d*_S_ = 3/0 [*P* = 0.004] and *d*_N_/*d*_S_ = 2/0 [*P* = 0.026], respectively); significantly, the canonical *b*-like Ser42Gly and Met95Ile substitutions occur along the stem (see Figure 2). The lineage leading to the clade that includes the colony LP719 allele as well as the *b*-like alleles was not identified as experiencing selection (*d*_N_/*d*_S_ = 1/0). A third branch under positive selection represents a relatively basal, well supported lineage within Clade IIIa consisting of one allele each from *S. invicta* and *S. macdonaghi* (*d*_N_/*d*_S_ = 4/1 [*P* = 0.003]).

When we applied the branch-site-specific (Bayes empirical Bayes random-effects) method to the ancestral branch subtending the LP719 allele and *b*-like clade, *d*_N_/*d*_S_ again did not differ significantly from one (*d*_N_/*d*_S_ = 1.05, *P* = 0.75). On the other hand, the method did detect a ratio significantly greater than one over the stem and interior branches of the *b*-like clade (*d*_N_/*d*_S_ = 17.5, *P*<0.00001). This approach identified with confidence two positions under positive selection on these *b*-like branches, 39 and 117, both of which also were identified by two of the site-specific methods applied over the entire tree (Table 4).

When we applied the branch-site-specific analysis to just the clade of *b* alleles (those *b*-like alleles bearing a charge-changing amino acid substitution at position 151), no significant signature of selection was detected on its stem lineage or internal branches.

**Figure 6:**
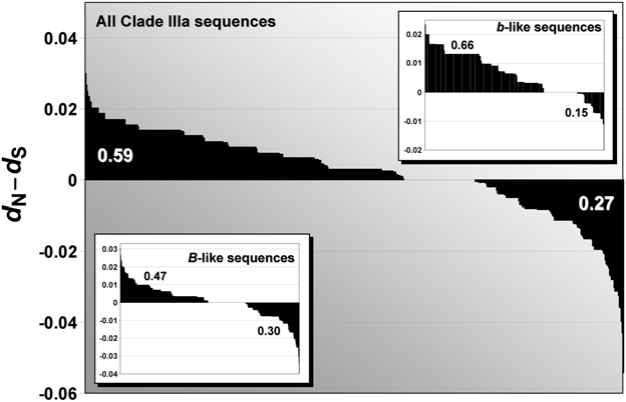
Distributions of overall pairwise estimates of *d*_N_-*d*_S_ for *Gp-9.* Estimates are shown separately for the set of all *Gp-9* sequences from the socially polymorphic South American fire ant species (Clade IIIa sequences) as well as for the subsets of all *B*-like and *b*-like sequences. Proportions of positive and negative *d*_N_-*d*_S_ values are indicated for each group of sequences.

Examination of overall *d*_N_-*d*_S_ estimates for each pair of *Gp-9* sequences from the socially polymorphic South American fire ants (Clade IIIa sequences) reveals a two-fold excess of positive values (Figure 6). Estimates obtained separately for the *B*-like and *b*-like classes show that the excess is especially marked for the latter class, consistent with evidence from several of the analyses above implicating positive selection in this clade of *Gp-9* alleles associated with polygyny.

### Phylogeography of *Gp-9* variants in S. *invicta*

Our sequence data confirm the occurrence of *b*-like alleles at all five sampling sites in the south-central portion of the native range previously reported to contain such alleles (based on allele-specific PCR analyses [26]). In addition, we discovered alleles of this class at four other sites well outside of their previously known area of occurrence (Corumba, Coxim, La Paz, and Suncho Corral; see Figure 1). These results suggest that while polygyny seems to be concentrated in northern Argentina and southeastern Brazil, it may occur at some frequency through much of the native range of *S. invicta*.

An initial analysis of molecular variance (AMOVA) of *B*-like alleles revealed that no detectable variation occurs among the ten arbitrarily clustered groups of sites once the 30% of total variation found among sites within groups is accounted for (significance of among-site differentiation; *P*<0.001). Similar results were obtained in a second analysis of 14 sites clustered according to patterns of regional differentiation at 14 neutral nuclear genes; no variation occurs among the regional groups, but 21% of the total variation occurs among sites within groups (*P* = 0.001 for among-site differentiation). For comparison with the results of this second *Gp-9* analysis, 14% of the variation at the neutral genes occurs among the regional groups, while 10% resides among sites within groups (*P*<0.001 for differentiation at both levels). Thus, *Gp-9* in the monogyne form of native *S. invicta* appears to exhibit somewhat stronger differentiation than neutral nuclear markers at very local scales but much weaker differentiation at broader geographic scales covering hundreds of kilometers.

No significant pattern of isolation-by-distance was detected using Nei's *D*_A_ values for the *B*-like alleles at 40 sites (one-tailed *P* = 0.087). Thus, differentiation in *Gp-9* composition does not increase in parallel with geographic separation of sites. This finding again contrasts with the strong isolation-by-distance patterns detected using neutral nuclear markers in native *S. invicta* populations [29].

## Discussion

The objective of this study was to survey naturally occurring molecular variation at *Gp-9*, a candidate gene of major effect on the expression of fire ant colony social organization, with a special emphasis on uncovering the extent and distribution of this variation in native populations of the well studied pest species *Solenopsis invicta*. Patterns of observed sequence variation at *Gp-*9 were examined with the following goals: i) to reconstruct the evolutionary relationships of variant sequences from *S. invicta* and its close relatives, ii) to identify important mutational factors affecting variation at the gene, iii) to examine the historical role of selection in shaping the variation, iv) to learn whether any single candidate amino acid residue in GP-9 protein is completely predictive of social organization, and v) to examine the geographical distribution of the observed variation. The motivation of the work was to help bridge the gap between functional biochemical information and molecular population genetic data by constructing a cogent evolutionary narrative of the genetic underpinnings of a major social adaptation (see [20]).

### General features of the molecular evolution of *Gp-9*

Our large survey of *Gp-9* sequences from native fire ant populations succeeded in uncovering a large number of unique variants, with over 120 alleles found in *S. invicta* alone. Intragenic recombination was inferred to be unimportant in generating the diversity of sequences observed, a conclusion reached also by Krieger and Ross [11].

The exon/intron structure of all 164 fire ant *Gp-9* variants is identical to that reported previously [10], [11], with one remarkable exception. A single *S. invicta* sequence contained a nonsynonymous nucleotide substitution in codon 154 that transformed it from a stop codon to a glutamine-encoding codon, thereby extending the C-terminal tail of the GP-9 protein by 22 amino acids. Two of these supernumerary residues are basic (Lys^159^, His^175^) while none is acidic, so that the mutant protein is likely to have a charge change mirroring or exceeding that of the proteins encoded by the distantly related *b* alleles of *S. invicta* (which have a unique Lys^151^ replacement). The charge change in the C-terminus of the *b*-encoded proteins is associated with recessive deleterious (lethal) effects not found in other *b*-like alleles [17]; these may stem from changes in the ligand binding/unloading properties or in the ability of the protein to form biologically active dimers, judging from the fact that the C-termini of odorant-binding proteins seem to be involved in these functions [30]. Demonstration of similar deleterious effects of the elongated mutant protein could pave the way for functional experiments intended to clarify some basic biochemical features of GP-9 protein.

Significant patterns in the nucleotide variation along *Gp-9* and its 3′ flanking region are evident when sequences from all the fire ant species are compared. First, the average amount of nucleotide variation in the exons exceeds that in the non-coding regions, based on the proportions of variable sites as well as the mean numbers of different nucleotides per site. Specifically, about one-fourth of coding-region nucleotide sites are variable but only half that proportion of non-coding sites are variable. The disparity persists regardless of whether all codon nucleotides or just third codon positions are considered, suggesting that most of the elevated exon nucleotide diversity consists of nonsynonymous substitutions. Superimposed on this general difference between coding and non-coding regions is considerable heterogeneity in the variation occurring within the two types of regions. Apparent elevation in variation above the background level for exons occurs in the 3′ portion of exon 5 (34% vs. 22% variable sites), much of which translates into elevated amino acid variation. Moreover, apparent depressions in variation below the background non-coding level occur in the middle of intron 2 and in the 3′-UTR (<6% vs. 14% variable sites).

Very similar patterns of variation along *Gp-9* are evident for just the sequences from the socially polymorphic species (Clade IIIa sequences) or from *S. invicta*. For *S. invicta*, the mean proportion of nucleotide substitutions (*p*) in the coding regions is elevated as much as 3-fold over that in non-coding regions, a difference judged to be highly statistically significant by a phylogeny-based resampling test (*P*<0.001). As is also true for the larger set of sequences, most coding-region nucleotide variation in *S. invicta* corresponds to amino acid replacements. With respect to absolute amounts of divergence over the entire gene region, only relatively modest nucleotide sequence differentiation occurs among the large number of *Gp-9* alleles recovered from nominal *S. invicta*. Most pairs within the *B*-like or *b*-like allele classes differ by fewer than a dozen point substitutions across the 2300 bp sequence alignment, and the most divergent alleles from this species differ at just 38 (1.6%) of their sites. The two most divergent sequences in the entire data set (from *S. aurea* and *S. invicta*) differ at just 47 (2.1%) of their sites.

The resulting picture of *Gp-9* sequence evolution in *S. invicta* and its fire ant relatives is that relatively few point mutations have accumulated across the gene, with only 15% of sites exhibiting variation, but a high proportion of these mutations occurred in the coding regions. Moreover, most of these coding-region substitutions led to amino acid replacements, so that in *S. invicta* alone over one-third of codons now encode variable residues. These findings raise several important points with respect to the molecular evolution of *Gp-9*. First, this pattern is consistent with a general lack of negative selection acting to constrain amino acid replacements over much of the encoded protein, as has been inferred also for other insect odorant-binding proteins based on their low amino acid sequence identities (the primary structure of these proteins evidently can be highly variable as long as the tertiary structure is conserved [18], [31]). Second, the elevated diversity observed in coding relative to non-coding regions may reflect different mutation rates or biases in the two types of sequence, which may in turn be related to the dramatically different base compositions [32]–[34]. Third, negative selection acting with greater overall force on the non-coding than coding regions may also play some role in the observed pattern of diversity; indeed, two non-coding tracts with extremely low variation across the surveyed sequences, including the entire 3′-UTR, potentially constitute evolutionarily constrained cis-regulatory elements [35], [36].

Finally, the elevated coding region nucleotide variation and high proportion of polymorphic amino acids might be construed as reflecting the historical action of positive selection, presumably an important general force in the evolution of insect odorant-binding proteins [37]. Direct evidence of some role for such selection on *Gp-9* in fire ants comes from our formal selection analyses. Several codons were identified by various site-specific methods as having significantly elevated rates of nonsynonymous over synonymous substitutions across the species, and two of these (at positions 39 and 117) were implicated as well by a branch-site-specific method applied to the *b*-like clade. Moreover, replacements at the latter two codons typically involved residues of different property groups. Position 117 also was identified by Krieger and Ross [11] as subject to positive selection based on a more diverse set of *Solenopsis Gp-9* sequences. Neither of these two consistently identified positions appears to be in the binding cavity or C-terminus of GP-9 protein based on earlier structure prediction analyses [11]. Thus, we cannot speculate about what physiological or other traits, if any, may be affected by the amino acid replacements at these locations.

More compelling evidence for positive selection having acted on *Gp-9* comes from the combined results of the branch-specific and branch-site-specific methods, as well as the overall *d*_N_-*d*_S_ estimates. These analyses consistently revealed elevated rates of amino acid replacement at the base of and throughout the *b*-like allele clade associated with polygyny in the socially polymorphic species. Similar results were obtained from the earlier selection analyses of Krieger and Ross [10], [11]. Congruent with these findings is our discovery of a highly elevated bias towards transversions at the third codon position in just the *b*-like lineage. The end result of this apparent burst of adaptive molecular evolution is a clade of rather similar alleles distinguished by low levels of silent substitutions but relatively high levels of amino acid replacements. Our study thus adds to the evidence that selection has played some creative role in the molecular evolution of *Gp-9* in fire ants, primarily in the context of the origin and elaboration of an alternative form of social behavior.

It is worth emphasizing that adaptive divergence of *Gp-9* generally does not seem to be associated with speciation events in the South American fire ants, a conclusion evident also from earlier studies [10], [11], [20]. This is inferred from the extensive paraphyly of *Gp-9* sequences with respect to the nominal species as well as the persistence of the *b*-like clade as a trans-species polymorphism. Together with the evidence that selection has promoted the divergence of the *b*-like clade, these patterns imply that intraspecific social evolution is a more important driver of *Gp-9* sequence diversification than is cladogenesis.

Two of the site-specific methods also yielded evidence of a single codon, at position 99, being under negative selection. This position is predicted to occur in the binding cavity of GP-9 [11], and so may represent a rare example where any variation in the encoded residue negatively impacts the binding capability and, hence, biological function of the protein.

### Association of *Gp-9* molecular variation with social organization

Among the coding-region diversity uncovered in our survey was substantial variation across the three codons that typically are jointly diagnostic between *b*-like and *B*-like alleles (positions 42, 95, 139) and, thus, predictive of colony social organization [10]. Rather than encoding alternate sets of amino acid residues at these three positions, the newly detected variants encode various combinations of *b*-like and *B*-like residues. Based on our determination of the social organization of the source colonies for these alleles, we conclude that no single residue is completely predictive of social behavior. Indeed, *b*-like residues invariably were present at all three positions in sequences from all polygyne colonies and, so, all of these residues may be jointly required for the expression of polygyny. Remarkably, placement of the novel variants in the *Gp-9* allele phylogeny indicates that both the Gly^42^ and Ile^95^ *b*-like residues arose independently on at least two occasions. Apparently, however, only when they appeared concurrently on the stem lineage of the *b*-like clade already bearing the Ile^139^ *b*-like residue did expression of polygyny become possible.

Our proposal that all three characteristic *b*-like replacements are completely associated with polygyne behavior in the socially polymorphic species and, by implication, potentially involved in its expression, contradicts the conclusion of Krieger [30] and Krieger and Ross [11] that Val139Ile was the lone crucial replacement. This conclusion was based primarily on the inference from protein structure modeling that residue 139 forms part of the ligand-binding cavity. However, the same analysis also predicted that residue 95 lies in the binding cavity, and residue 42, although not expected to function in ligand binding, experienced a replacement between amino acids of different property groups. Although no study to date has identified codon 139 as being under positive selection, the Bayesian random-effects test implemented in this study implicated such selection on both codons 42 and 95. Moreover, our branch-specific analyses implicated positive selection on the stem lineage of the *b*-like clade, where the Ser42Gly and Met95Ile replacements occurred, but not on the preceding ancestral branch where the Val139Ile replacement occurred. We note that our data do not completely rule out the possibility that some combination of only two *b*-like residues may underlie polygyny, but testing this possibility must await discovery of colonies that contain such variants but lack typical *b*-like sequences.

### Geographic distribution of *Gp-9* molecular variation

A previous survey of *Gp-9* polymorphism in *S. invicta* using allele-specific PCR indicated that the *b*-like allele clade (and, by extension, polygyny) is restricted to the south-central portion of this species' range [26]. Our far more extensive sampling has extended the known area over which these alleles occur considerably northward into central Brazil. Nonetheless, in view of the fact that the ranges of the other socially polymorphic species harboring *b*-like alleles are restricted to eastern Argentina, Uruguay, and southeastern Brazil [38], an origin of the *b*-like clade in this area seems likely.

We found no indication of significant higher-level (regional) structure in the geographic distribution of *B*-like variants of *Gp-9* in *S. invicta*, although local populations are highly differentiated from one another. This pattern stands in contrast to the striking regional differentiation observed for numerous other, presumably neutral, nuclear loci. One possible explanation for the difference is that, by chance, the distribution of *Gp-9* variation does not closely track that of the remaining nuclear genome simply because of the probabilistic nature of allele lineage sorting (e.g., [39]). Another possibility is that sporadic interspecific hybridization in different areas followed by introgression of heterospecific *Gp-9* alleles has broken down any geographic pattern that may have developed due to restricted inter-regional gene flow. Several lines of evidence support the plausibility of this scenario. First, the *B*-like alleles of *S. invicta* are extensively paraphyletic or polyphyletic with respect to the alleles of several other fire ants, including some species regarded as quite distant relatives [24]. Second, the *S. invicta* sequences that are polyphyletic with respect to these distant relatives often were obtained from colonies located within or close to the range of the other species (e.g., the closely related *S. invicta* and *S. saevissima* alleles in Clade II and the closely related *S. invicta*, *S. saevissima*, and *S. altipunctata* alleles in the sister lineage of Clade IIIA; see Figure 4). Finally, parallel patterns of minimal regional differentiation coupled with interspecific sequence paraphyly and polyphyly have been observed for the mtDNA of *S. invicta* [29], [40], with the latter features almost certainly the result of introgression.

This scenario posits that *Gp-9* (and the mtDNA) flows more freely between fire ant species (and, perhaps, among regional *S. invicta* populations) than the bulk of the nuclear genome, perhaps because of a lack of selection against these introgressing elements (or, in the case of the mtDNA, because of selection favoring the spread of the cytoplasmic symbiont *Wolbachia* [41]). One important implication of this scenario, if true, is that polygyny may not have arisen in the common ancestor of the socially polymorphic fire ants, with the *b*-like lineage persisting through multiple speciation events [10], [11]. Rather, this allele class and the alternate form of social behavior with which it is associated conceivably arose more recently, then spread among species of the socially polymorphic clade through hybridization (see also [42]).

### Conclusions

We recovered numerous *Gp-9* alleles from *S. invicta* and other South American fire ants in their native ranges. Relatively little overall variation distiguishes these alleles, but the distribution of the variation along the gene and within the gene phylogeny is noteworthy. A surprising amount is found in the coding regions of the gene, with substitutions there usually causing amino acid replacements. Indeed, the proportion of variable amino acid positions is more than twice the proportion of variable nucleotide sites over the entire gene region, both across species and within *S. invicta*. The elevated coding-region variation may result from a general lack of negative selection acting to constrain amino acid replacements, different mutation rates or biases in coding and non-coding regions, negative selection acting with greater force on non-coding than coding regions, or most likely, positive selection acting on the protein in the *b*-like allele clade associated with polygyny. Finally, our determination of the social organization of key colonies confirmed the invariant link between the presence of typical *b*-like alleles and expression of polygyny, while our discovery of several novel alleles bearing various combinations of *b*-like and *B*-like codons revealed that no single amino acid residue is completely predictive of polygyne behavior.

This study thus yields information of use in bridging population genetic and functional approaches to understanding the genetic basis of polygyny in fire ants. With the inception of a broad, integrative approach to investigating the biochemical pathways in which the *Gp-9* product functions, the phenotypic effects of molecular variation at *Gp-9* and other pathway genes, and the potential involvement of other genes in linkage disequilibrium with *Gp-9*, substantial progress toward understanding the evolution of this key social adaptation can be expected.

## Materials and Methods

### Sampling

We obtained samples from several *Solenopsis* species of varying phylogenetic relationship to *S. invicta* [24] to assess the nature and extent of *Gp-9* sequence variation in fire ants and to evaluate the monophyly of *S. invicta* sequences with respect to those of its closest relatives. These samples consisted of a single individual per nest collected in Argentina, Brazil, and the western USA between 1992 and 2001. Samples of the following species were obtained (numbers of individuals [nests] in parentheses): *S. altipunctata* (1), *S. amblychila* (1), *S. aurea* (1), *S. daguerrei* (2), *S. electra* (1), *S. interrupta* (4), *S. macdonaghi* (4), *S. megergates* (6), *S. pusillignis* (3), *S. quinquecuspis* (6), *S*. *richteri* (8), *S. saevissima* (9), *S. xyloni* (1), and the undescribed *S.* species “X” (2).

Samples of nominal *S. invicta* were obtained from 132 nests from 44 sites distributed over much of the native South American range (Figure 1) as well as from two sites in the introduced range in the USA (Georgia and California) between 1988 and 2004. Sites in the native range were chosen not only to maximize geographic coverage but also to include all of the genetically differentiated populations distinguished in earlier studies of neutral nuclear and mtDNA variation [29], [40], [43]. The purpose of this targeted sampling scheme was to uncover the maximal amount of diversity at *Gp-9* in the focal species in order to generate a complete sequence phylogeny, which in turn formed the framework for our formal selection analyses. Multiple nests were sampled at many of the sites (see Figure 1), but generally only a single specimen was used from any single nest. The social organization of many of the sampled *S. invicta* colonies was determined previously by a combination of methods including discovery of multiple reproductive queens, determination of the number of offspring matrilines using allozyme markers, and detection of *b*-like *Gp-9* alleles [26], [43], [44].

Live specimens of all the study species were collected directly from nests in the field then placed immediately on liquid nitrogen for transport back to the laboratory, where they were held in a −80°C freezer pending genetic analysis.

### Sequencing of *Gp-9*

Sequencing methods followed the protocols of Krieger and Ross [10], [11], with some modifications. DNA was extracted using the Puregene DNA Isolation Kit (Gentra Systems, Minneapolis, MN). Polymerase chain reaction (PCR) reactions were set up in 10 µl volumes using 1.1× high fidelity PCR-ready reaction mix (Bio-X-Act Short Mix, Bioline, Randolph, MA) and 0.2 µM primers, with a hotstart thermal cycling regime starting at 95°C and followed by 35 cycles at 94°C (20 s), 62°C (30 s), and 68°C (1 min 40 s), and with a final elongation step at 68°C (10 min). Primer sequences were those used by Krieger and Ross [10] (Gp-9/-33 forward: 5′-CATTCAAAGTACAGTAGAATAACTGCC-3′, Gp-9_2218 reverse: 5′-CAGGAGTTTGAGTTTGTCACTGC-3′). The approximately 2200-bp amplification products included the full length 1700-bp *Gp-9* gene (containing five exons and four introns) as well as a 500-bp segment of the 3′ flanking region containing the 170-bp UTR. These products were gel purified (QIAquick Gel Extraction Kit, Qiagen, Valencia, CA) and cloned into pCR2.1 vectors (Invitrogen, Carlsbad, CA), which were then used to transfect competent TOP10F′ *E. coli* cells (Invitrogen). Blue-white screening was used to identify positive clones, which were picked and subjected directly to a hotstart PCR amplification using M13 or the *Gp-9* primers (same conditions as in the previous PCR but using Taq-Pro Complete, Denville Scientific Inc., Meutchen, NJ). The resulting PCR product was checked for correct length by running it out on an ethidium bromide-stained agarose gel, then it was purified using PEG 8000 (Promega, Madison, WI).

Methods for conducting DNA sequencing reactions using internal primers also followed the protocols of Krieger and Ross [10], [11]. Reactions were performed using the ABI PRISM BigDye Terminator v3.1 Cycle Sequencing Kit (Applied Biosystems, Foster City, Calif.), with the products run out in an ABI PRISM 3740xl DNA Sequencer (Applied Biosystems). In light of the considerable overlap of the internal sequencing reads, sequences were not determined in the reverse directions. Critical base calls in phylogenetically important sequences were confirmed by resequencing.

In order to ensure a sufficiently large sample of *b*-like alleles, clones derived from suspected polygyne *S. invicta* colonies were screened for such alleles using competitive allele-specific PCR [45]. Reaction mixes contained 0.13 µM primers, 0.33 µM complementary primer, 1× Taq-Pro Complete, and 0.5 µL of the clone PCR product; PCR was conducted using a cycling regime of 94°C (2 min), 35 cycles at 94°C (45 s), 64°C (45 s), and 72°C (1 min), and with a final elongation step at 72°C (5 min). The primers used in this allele-specific PCR recognize the single nucleotide substitutions at codons 95 and 139 considered to be jointly diagnostic of all *b*-like alleles [10], [21].

### Determination of colony social organization

Most colonies of unknown social organization yielding sequences that encoded one or more residues considered diagnostic for *b*-like alleles (Gly^42^, Ile^95^, Ile^139^) were subjected to microsatellite analyses to learn whether these colonies were monogyne or polygyne. Genotypes at eight loci (*Sol-6*, *Sol-11*, *Sol-18*, *Sol-42*, *Sol-49*, *Sol-55*, *SolM-III*, and *SolM-V*) were scored for 7–12 workers from each nest (PCR methods in [46]–[48]). PCR products were visualized using an ABI PRISM 3740xl DNA Sequencer. Queens of native *S. invicta* normally mate only once [44], [49], so the presence of more than three alleles at a locus among a colony's workers indicates the presence of multiple offspring matrilines (polygyny).

### Genetic analyses

All *Gp-9* sequences were readily aligned by hand. The aligned sequences were tested for evidence of recombination using the DSS and LRT methods implemented in the program TOPALI [50].

Differentiation among *Gp-9* alleles in their nucleotide composition was tested using homogeneity χ^2^ analysis (implemented in the program PAUP* [51]) and visual inspection (implemented in the program SeqVis [52]). Non-random codon usage was tested using the program DNASP [53], with Yates' correction for the observed G+C content employed.

Transition and transversion rates were plotted against Tamura-Nei (TN93) distances for all pairs of sequences in various subsets of the data using the program DAMBE [54] to look for evidence of mutational saturation. In addition, the extent of transition bias (transition/transversion rate ratio) was estimated for the combined first and second codon positions and for the third codon position of exons in various data subsets using the program MEGA [55].

Several measures of nucleotide sequence variation were estimated for the *Gp-9* alleles of nominal *S. invicta*. The uncorrected mean number of nucleotide substitutions (*d*) and proportion of nucleotide substitutions (*p*) were calculated for the coding regions (all positions and third codon positions) and the non-coding regions of all pairs of unique alleles [56] using MEGA. To measure the levels of observed nucleotide and amino acid variation in the coding regions of *S. invicta* sequences, we estimated two additional diversity indices for all unique sequences, the codon diversity and amino acid diversity [11]. Codon diversity denotes the variation at each in-frame coding-region triplet, while amino acid diversity denotes the variation at the corresponding amino acid residue. Values for the two measures range from zero to one for a given codon; a value of zero signifies a location with identical codons (or amino acids) in all sequences, whereas a value of one indicates that every unique sequence displays a unique codon (or amino acid) at this location. These indices were compared to determine the extent to which observed coding-region nucleotide variation translates into amino acid replacements. All of these analyses were performed separately for the *b*-like alleles, *B*-like alleles, and all alleles combined. Other measures of sequence variation that incorporate haplotype frequency estimates (such as π) were not estimated because our targeted sampling scheme would not yield unbiased frequency estimates.

We tested for differences in substitution rates between coding and non-coding regions of *S. invicta* sequences using two statistical approaches. First, a Fisher's exact test compared *p* between the two partitions. Second, a resampling test was used to compare tree lengths between coding and non-coding regions. A null distribution of tree lengths of non-coding nucleotides was generated by randomly resampling (without replacement) 462 such nucleotides from the original data matrix 1,000 times using MESQUITE [57], then calculating tree lengths for each resampled matrix on the Bayesian inference (BI) phylogeny under both maximum parsimony (MP) and maximum likelihood (ML) criteria using PAUP*. Tree lengths computed for the 462-bp coding region under these criteria on the same BI tree were compared to the tree lengths derived from the randomly resampled non-coding characters.

### Analyses of amino acid replacements

Patterns of amino acid variation at GP-9 were depicted graphically by generating sequence logos [58] using the program WEBLOGO [59]. Symbols were color-coded based on the chemical properties of each amino acid according to the scheme of Parry-Smith et al. [60]. Amino acid replacements were mapped onto the *Gp-9* phylogeny using parsimony reconstruction as implemented in the program MACCLADE [61].

### Phylogenetic analyses

Sequences from the North American fire ant species *S. amblychila*, *S. aurea*, and *S. xyloni* were specified as outgroups for all phylogenetic analyses (see [24]). We assessed the potential impact on the phylogenetic analyses of heterogeneity in sequence nucleotide composition by constructing preliminary phylogenies with the Neighbor-Joining method [62], using the minimum evolution (ME) criterion based on either LogDet [63] or the ML distances between alleles (with all parameters estimated from the data). An SH test [64] conducted on the resulting two trees revealed no evidence of compositional heterogeneity (Δ-lnL = 7.543, *P* = 0.397).

Due to the prohibitive computational time required for even a single heuristic search under the MP criterion, we employed the parsimony ratchet method [65]; this approach was implemented by means of the program PAUPRAT [66] using 500 repetitions and randomly perturbing 25% of the characters for each re-weighting. The analysis was repeated ten times to ensure that tree space had been adequately searched, then repeated another ten times while considering gaps as character states.

Because the MP and ME phylogenies did not differ significantly according to an SH test (Δ-lnL = 112.3, *P* = 0. 124), we estimated the best fitting model of *Gp-9* nucleotide evolution from the better resolved ME tree using the program MODELTEST [67]. We selected the most appropriate models for the complete data set and various partitions of it (non-coding regions; coding regions; first, second, and third codon positions) using the Akaike information criterion [68] and Bayesian information criterion [69] (see [70]).

Finally, we conducted four independent Markov chain Monte Carlo (MCMC) tree searches under the BI optimality criterion using the program MRBAYES [23], [71]. Multiple analyses were run to ensure adequate exploration of tree and parameter space [23], [72]. Five parallel chains, four of which were heated incrementally (temperature = 0.1), were started from random trees for each analysis, with initial parameter values based on the evolutionary model selected by MODELTEST. The chains were run for two million generations, with sampling every 100 generations. Stationarity of the chains was ascertained visually by plotting sample log-likelihoods through the course of each run, as well as by examining the convergence diagnostics (the potential scale reduction factor for all parameters approached 1.0 at stationarity) [23]. Pre-stationarity MCMC samples were discarded as burn-in (usually, around the first 700 samples), and the model parameters and tree topology were estimated using the remaining samples. The log-likelihoods, substitution models, and tree topologies were compared among independent runs using SH tests. After ensuring that all runs had converged on the same area in tree/parameter space, the samples from the four different runs were combined for final analysis.

### Selection analyses

Two general approaches for comparing nonsynonymous and synonymous substitution rates were employed to test for positive selection on *Gp-9* in our complete data set, random-effects and counting analyses [27]. Fixed-effects analyses were not employed because they require a priori designation of sites evolving under different selective regimes [27]; we intended to use our data set for a largely independent test of the findings of Krieger and Ross [10], [11] and so wished to avoid biasing the results by focusing on specific sites previously identified as being under selection.

We employed two different random-effects methods, which fit a distribution of substitution rates across sites and then infer the rate at which each site evolves [73]. Because of computational limitations, the Bayesian method [74] was conducted on a reduced data set (25 exemplar sequences representing all major clades in the BI phylogeny) using the program MRBAYES. Coding and non-coding sites were separated into unlinked partitions for the analysis. Selection on the coding partition was estimated according to the M3 codon model [75], which is less restrictive than the commonly used Nielsen and Yang model [73] (see [23], [71]). MCMC chains were run for one million generations, with sampling every 100 generations. Otherwise, model specifications followed those used in the BI phylogeny estimation.

The second random-effects method used was a maximum likelihood branch-site-specific model [76], [77]. We removed all non-coding sites from the data set, then used the BI phylogeny as a guide to prune sequences that were sisters to sequences with identical coding regions or occurred in unresolved clades of identical coding sequences (a single exemplar was retained). This resulted in a reduced data set of 92 sequences. The CODEML program in the PAML package [78] was used to run the branch-site-specific model A, test 2 [77], which infers selection on codons along specified branches. We used the pruned BI phylogeny (with branch lengths estimated under the M3 model) to test for selection on the stem lineages and internal branches of the *b*-like allele clade as well as the *b* allele clade (the smaller clade of *b*-like alleles featuring the Glu151Lys substitution). We adopted the Bayes empirical Bayes [79] approach in place of the naive empirical Bayes approach to identify sites under selection [80]. The analysis incorporated the F3×4MG model of codon substitution [81] because it yielded better likelihood scores than the F3×4NY model [73]. We ran each analysis three times using different initial values for the parameters ω and κ.

We employed three different counting methods based on the Suzuki-Gojobori approach [82]. These methods estimate the number of nonsynonymous and synonymous substitutions at each codon position, then test for significant differences between the number of nonsynonymous changes per nonsynonymous site (*d*_N_) and number of synonymous changes per synonymous site (*d*_S_). The reduced data set of 92 sequences created for the branch-site-specific random-effects method was used also for the counting methods. The first counting method was the single likelihood ancestor counting (SLAC) analysis implemented in the program DATAMONKEY [83]. The global *d*_N_/*d*_S_ ratio was estimated along with 95% confidence intervals using the HKY model of nucleotide substitution, with ambiguous characters resolved according to the most likely solution given by the model.

The second counting method we used follows more closely the original Suzuki and Gojobori [82] approach. This analysis was conducted in association with the branch-site-specific random-effects analysis using CODEML.

As a third counting method for detecting selection, we used the Zhang et al. method [28] adapted from the approach of Messier and Stewart [84]. This approach uses reconstructed ancestral sequences to test the null hypothesis of neutral evolution (*d*_N_ = *d*_S_) along each branch of the inferred phylogeny by means of Fisher's exact tests. To maximize the statistical power of this test, we pooled non-coding sites with coding-region synonymous sites (e.g., [85]) after determining that there was no significant difference in substitution rates between these partitions (Fisher's exact test on *p*, *P* = 0.115). Ancestral sequences were reconstructed using the BASEML program in PAML, with *d*_N_ and *d*_S_ for each branch estimated using the “free-ratio” model [73]. We did not test for negative selection using this method.

Finally, we calculated maximum likelihood estimates of *d*_N_ and *d*_S_ for each pair of *Gp-9* sequences from the socially polymorphic South American fire ants using CODEML to look for evidence of broad positive selection. These analyses also were conducted separately for the *B*-like and *b*-like alleles.

Tests such as the McDonald-Kreitman and HKA tests that compare intraspecific variation with interspecific divergence in order to detect signals of positive selection were not employed in this study because the major bouts of adaptive divergence in *Gp-9* appear to be associated with social evolution within species rather than with speciation events. Also, tests using site-frequency spectrum data to test for deviations from neutral theory predictions (e.g., Tajima *D* test, Fay and Wu *H* test) were not employed because our targeted samples cannot provide the unbiased estimates of haplotype frequencies required for their proper application [86].

### Phylogeographic analyses

Evidence for geographical partitioning of related *Gp-9* alleles in native *S. invicta* was examined by conducting a series of analysis of molecular variance (AMOVA) analyses [87] using the program ARLEQUIN [88]. This procedure partitions total genetic variation among the different sampling sites or clusters of sites in order to reveal hierarchical patterns of spatial differentiation. Only *B*-like alleles were included in order to avoid any effect of spatial restriction of polygyny (e.g., [26]). Genetic distances between *Gp-9* alleles were estimated as the squares of the numbers of pairwise sequence differences. In an initial analysis, all 40 sites containing at least one *B*-like allele were clustered arbitrarily into ten regional groups (these groups occupied areas 100–300 km in diameter). In a second analysis, only the 14 sites for which three or more sequences were available were used. In this case, goups of sites were clustered on the basis of patterns of regional differentiation previously detected at 14 presumed neutral nuclear loci [29] (these groups occupied areas 100–600 km in diameter). Parallel AMOVA analyses using the neutral nuclear data of Ross et al. [29] from the same 14 sites were conducted to provide a direct comparison with the *Gp-9* results from the second analysis. All alleles at each neutral locus were assumed to be equally related to one another (i.e., an infinite alleles model of mutation was assumed). Statistical significance of genetic differentiation among sites or clusters of sites was determined by permuting alleles across individuals (20,000 replicates) for *Gp-9* or bootstrapping over loci (10,000 replicates) for the neutral markers.

Isolation-by-distance analyses were conducted for the *Gp-9* sequences of *S. invicta* to learn whether differentiation between sites in their allele composition increases in parallel with their geographic separation. Only the 40 sites from which one or more *B*-like alleles were sampled were considered. The program GENEPOP [89] was used to examine the relationship of Nei's net number of nucleotide differences (*D*_A_ [90]) with the natural logarithm of geographic distances between sites (see [91], [92]). Significance of isolation-by-distance relationships was determined by means of Mantel tests based on 10,000 data permutations coupled with estimation of Spearman rank correlation coefficients.

## Supporting Information

Table S1 GenBank Accession Numbers for *Gp-9* Sequences Used in this Study.
(0.06 MB DOC)
