# Supplementary material for: Molecular Variation at a Candidate Gene Implicated in the Regulation of Fire Ant Social Behavior
Source: PLoS One. 2007 Nov 7;2(11):e1088. doi: 10.1371/journal.pone.0001088 (PMC2043488; doi:10.1371/journal.pone.0001088)
Supplement: Supplementary file 1 [file pone.0001088.s001.doc]

## Sheet: Table S1

Table S1.  GenBank accession numbers for Gp-9 sequences used in this study.
	Colony /
Species	sample code	Sample collection site	Accession number
Solenopsis amblychila	SC1	Portal, Arizona, USA	AF427889
S. aurea	JPAZ2	Portal, Arizona, USA	AF427890
S. xyloni	JPAZ4	Portal, Arizona, USA	AY818623
S. pusillignis	Pi-62	Corumba, Brazil	EU220032
S. pusillignis	Pi-66c	Corumba, Brazil	EU220033
S. pusillignis	Pi-66	Corumba, Brazil	AY818624
S. electra	AR-26a	Santiago del Estero, Argentina	AY818625
S. daguerrei	Pu-35dag	Makalie, Argentina	AY818626
S. daguerrei	W-14dag	Nazaré, Brazil	EU220034
S. altipunctata	G-86	Santa Cecilia, Brazil	AY818627
S. interrupta	Pu-42/a	Villa San Martin, Argentina	EU220035
S. interrupta	Pu-42/b	Villa San Martin, Argentina	EU220036
S. interrupta	Pu-46	Morrison, Argentina	EU220037
S. interrupta	AR-28b	Ojo de Agua, Santiago del Estero, Argentina	AF427891
S. saevissima	O-1	Belo Horizonte, Brazil	AF427892
S. saevissima	O-3m	Belo Horizonte, Brazil	EU220038
S. saevissima	W-14	Nazare, Brazil	AF427892
S. saevissima	W-31	Fortaleza, Brazil	EU220039
S. saevissima	W-42	Doca, Brazil	EU220040
S. saevissima	W-44	Capanema, Brazil	AY818638
S. saevissima	W-72	Posse, Brazil	EU220041
S. saevissima	G-82	Rio Negro, Brazil	AY818636
S. saevissima	W-84	Aqua Clara, Brazil	AY818637
S. quinquecuspis	Pu-100	Rosario, Argentina	EU220042
S. quinquecuspis	Pu-60m	Rosario, Argentina	EU220043
S. quinquecuspis	Pu-59m	Rosario, Argentina	EU220044
S. quinquecuspis	Pu-49	Rosario, Argentina	AF427896
S. quinquecuspis	Pu-49	Rosario, Argentina	AF427902
S. quinquecuspis	B-16	Rosario, Argentina	AY818633
S. species "X"	AR-44a	Buenos Aires, Argentina	EU220045
S. species "X"	AR-35	Canada de Gomez, Argentina	AY818635, AY818634
S. richteri	Pu-72c	Rosario, Argentina	EU220046
S. richteri	Pu-13p/c	Arroio dos Ratos, Brazil	EU220047
S. richteri	Pu-77c	Rosario, Argentina	EU220048
S. richteri	R5-R/c	Rosario, Argentina	EU220049
S. richteri	Pu-13/o	Arroio dos Ratos, Brazil	EU220050
S. richteri	R15-R	Rosario, Argentina	AF427903
S. richteri	R15-R	Rosario, Argentina	AF427904
S. richteri	Pu-13f	Arroio dos Ratos, Brazil	AY818628
S. megergates	G-77/a	Palmeira, Brazil	EU220051
S. megergates	G-77/b	Palmeira, Brazil	EU220052
S. megergates	G-79	Curitiba, Brazil	EU220053
S. megergates	G-98	Arroio dos Ratos, Brazil	AY818632
S. megergates	G-98	Arroio dos Ratos, Brazil	AY818630
S. megergates	G-98	Arroio dos Ratos, Brazil	AY818629
S. macdonaghi	Pi-80	Itaquirai, Brazil	EU220054
S. macdonaghi	Pu-23	Itati, Argentina	AF427895
S. macdonaghi	Pu-23	Itati, Argentina	AF427901
S. macdonaghi	Pu-37	Quimili, Argentina	EU220055
S. invicta	Hickman-2	Hickman, California, USA	AF427898
S. invicta	MJBK-99	Eatonton, Georgia, USA	AF427897
S. invicta	Hickman-2	Hickman, California, USA	AF427894
S. invicta	Hickman-3	Hickman, California, USA	AF427893
S. invicta	Pu-14f	Arroio dos Ratos, Brazil	EU220056
S. invicta	Pu-14a	Arroio dos Ratos, Brazil	EU220057
S. invicta	Pu-14c	Arroio dos Ratos, Brazil	EU220058
S. invicta	Pu-14r	Arroio dos Ratos, Brazil	EU220059
S. invicta	Pu-15c	Arroio dos Ratos, Brazil	EU220060
S. invicta	Pu-15e	Arroio dos Ratos, Brazil	EU220061
S. invicta	Pu-15a/c	Arroio dos Ratos, Brazil	EU220062
S. invicta	Pu-11h/c	Arroio dos Ratos, Brazil	EU220063
S. invicta	Pu-15l	Arroio dos Ratos, Brazil	EU220064
S. invicta	BQ-347	Barranqueras, Argentina	EU220065
S. invicta	BQ-348	Barranqueras, Argentina	EU220066
S. invicta	BQ-361	Barranqueras, Argentina	EU220067
S. invicta	BQ-366	Barranqueras, Argentina	EU220068
S. invicta	BQ-367	Barranqueras, Argentina	EU220069
S. invicta	Pi-71-nmq-15	Campo Grande, Brazil	EU220070
S. invicta	Pi-72-nmq-24	Campo Grande, Brazil	EU220071
S. invicta	Pi-72-nmq-27	Campo Grande, Brazil	EU220072
S. invicta	Pi-72-nmq-27	Campo Grande, Brazil	EU220073
S. invicta	Pi-94	Ceu Azul, Brazil	EU220074
S. invicta	G-1c	Ceu Azul, Brazil	EU220075
S. invicta	G-3	Ceu Azul, Brazil	EU220076
S. invicta	G-28/c	Ceu Azul, Brazil	EU220077
S. invicta	G-28	Ceu Azul, Brazil	EU220078
S. invicta	G-42	Ceu Azul, Brazil	EU220079
S. invicta	G-68f	Ceu Azul, Brazil	EU220080
S. invicta	CL-308	Clorinda, Argentina	EU220081
S. invicta	CL-334	Clorinda, Argentina	EU220082
S. invicta	CL-336	Clorinda, Argentina	EU220083
S. invicta	CL-338	Clorinda, Argentina	EU220084
S. invicta	P1-6	Corrientes, Argentina	EU220085
S. invicta	P1-61	Corrientes, Argentina	EU220086
S. invicta	P1-72	Corrientes, Argentina	EU220087
S. invicta	P1-61	Corrientes, Argentina	EU220088
S. invicta	P1-65	Corrientes, Argentina	EU220089
S. invicta	P1-67	Corrientes, Argentina	EU220090
S. invicta	P1-81	Corrientes, Argentina	EU220091
S. invicta	X-1	Corrientes, Argentina	EU220092
S. invicta	Pi-26	Coxim, Brazil	EU220093
S. invicta	Pi-27	Coxim, Brazil	EU220094
S. invicta	Pi-29	Coxim, Brazil	EU220095
S. invicta	Pi-28	Coxim, Brazil	EU220096
S. invicta	P2-8	Formosa, Argentina	AF427899
S. invicta	P2-15	Formosa, Argentina	AF459414
S. invicta	P2-35	Formosa, Argentina	EU220097
S. invicta	P2-36	Formosa, Argentina	AF427899
S. invicta	P2-41	Formosa, Argentina	EU220098
S. invicta	P2-44	Formosa, Argentina	EU220099
S. invicta	P2-46	Formosa, Argentina	AF427900
S. invicta	P2-50	Formosa, Argentina	EU220100
S. invicta	P2-52	Formosa, Argentina	AF459414
S. invicta	P2-57	Formosa, Argentina	EU220101
S. invicta	P2-57	Formosa, Argentina	EU220102
S. invicta	P2-58	Formosa, Argentina	AF427900
S. invicta	P2-66	Formosa, Argentina	EU220103
S. invicta	P2-68	Formosa, Argentina	EU220104
S. invicta	P2-68	Formosa, Argentina	EU220105
S. invicta	P2-72	Formosa, Argentina	EU220106
S. invicta	Pu-31	Formosa, Argentina	EU220107
S. invicta	LP-719	La Paz, Argentina	EU220108
S. invicta	LP-728	La Paz, Argentina	EU220109
S. invicta	LP-720	La Paz, Argentina	EU220110
S. invicta	LP-729	La Paz, Argentina	EU220111
S. invicta	LP-745/a	La Paz, Argentina	EU220112
S. invicta	LP-745/b	La Paz, Argentina	EU220113
S. invicta	O-23	Pedra Preta, Brazil	EU220114
S. invicta	O-40	Pedra Preta, Brazil	EU220115
S. invicta	O-70	Pedra Preta, Brazil	EU220116
S. invicta	G-72	Pedra Preta, Brazil	EU220117
S. invicta	O-98	Pontes E Lacerda, Brazil	EU220118
S. invicta	Pi-3/a	Pontes E Lacerda, Brazil	EU220119
S. invicta	Pi-3/f	Pontes E Lacerda, Brazil	EU220120
S. invicta	Pi-10f	Pontes E Lacerda, Brazil	EU220121
S. invicta	Pi-12c	Pontes E Lacerda, Brazil	EU220122
S. invicta	Pu-7c	Rinco dos Cabrais, Brazil	EU220123
S. invicta	Pu-8v	Rinco dos Cabrais, Brazil	EU220124
S. invicta	Pu-9f	Rinco dos Cabrais, Brazil	EU220125
S. invicta	Pu-9k	Rinco dos Cabrais, Brazil	EU220126
S. invicta	B-23	Rosario, Argentina	EU220127
S. invicta	B-26/a	Rosario, Argentina	EU220128
S. invicta	B-26/b	Rosario, Argentina	EU220129
S. invicta	B-26/c	Rosario, Argentina	EU220130
S. invicta	B-32	Rosario, Argentina	EU220131
S. invicta	B-33	Rosario, Argentina	EU220132
S. invicta	B-33/a	Rosario, Argentina	EU220133
S. invicta	B-33/f	Rosario, Argentina	EU220134
S. invicta	B-43/f	Rosario, Argentina	EU220135
S. invicta	B-43	Rosario, Argentina	EU220136
S. invicta	B-47	Rosario, Argentina	EU220137
S. invicta	C-20	Rosario, Argentina	EU220138
S. invicta	Pu-49c	Rosario, Argentina	EU220139
S. invicta	SC-643	San Carlos, Argentina	EU220140
S. invicta	SC-645	San Carlos, Argentina	EU220141
S. invicta	SC-665	San Carlos, Argentina	EU220142
S. invicta	SC-665	San Carlos, Argentina	EU220143
S. invicta	SC-668	San Carlos, Argentina	EU220144
S. invicta	SC-670	San Carlos, Argentina	EU220145
S. invicta	AR-25b	Santiago del Estero, Argentina	EU220146
S. invicta	Pu-39	Santiago del Estero, Argentina	EU220147
S. invicta	Pu-40	Santiago del Estero, Argentina	EU220148
S. invicta	AR-25e	Santiago del Estero, Argentina	EU220149
S. invicta	Pi-47	São Gabriel do Oeste, Brazil	EU220150
S. invicta	Pi-59	São Gabriel do Oeste, Brazil	EU220151
S. invicta	Pi-59/c	São Gabriel do Oeste, Brazil	EU220152
S. invicta	Pi-59/f	São Gabriel do Oeste, Brazil	EU220153
S. invicta	AR-9e	Coronda, Argentina	EU220154
S. invicta	AR-13d	San Justo, Argentina	EU220155
S. invicta	AR-17f	Las Toscas, Argentina	EU220156
S. invicta	G-78	Curitiba, Brazil	EU220157
S. invicta	G-80	Rio Negro, Brazil	EU220158
S. invicta	G-89	Correia Pinto, Brazil	EU220159
S. invicta	G-93	Vacaria, Brazil	EU220160
S. invicta	G-94	Nova Petropolis, Brazil	EU220161
S. invicta	G-95	Pôrto Alegre, Brazil	EU220162
S. invicta	O-78	São Pedra da Cipa, Brazil	EU220163
S. invicta	O-86	Mirasol d'Oeste, Brazil	EU220164
S. invicta	O-88	Pôrto Esperidião, Brazil	EU220165
S. invicta	Pi-21	Comodoro, Brazil	EU220166
S. invicta	Pi-25	Rondonópolis, Brazil	EU220167
S. invicta	Pi-71	Miranda, Brazil	EU220168
S. invicta	Pi-74	Nova Alvorada do Sul, Brazil	EU220169
S. invicta	Pi-78	Carapó, Brazil	EU220170
S. invicta	Pi-84	Guaira, Brazil	EU220171
S. invicta	Pu-16	Soledade, Brazil	EU220172
S. invicta	Pu-17	Lajeado do Bugre, Brazil	EU220173
S. invicta	Pu-18	São Miguel d'Oeste, Brazil	EU220174
S. invicta	Pu-21	Ita Ibate, Argentina	EU220175
S. invicta	Pu-30	Velaz, Argentina	EU220176
S. invicta	Pu-36	Charada, Argentina	EU220177
S. invicta	Pi-65	Corumba, Brazil	EU220178
S. invicta	Pu-34	Resistencia, Argentina	EU220179
S. invicta	Pu-38	Suncho Corral, Argentina	EU220180
S. invicta	Pu-20	Posadas, Argentina	EU220181
sequences newly generated for this study are indicated in bold; b-like alleles are indicated with underlining.
